# Supplementary material for: Serum-free differentiation platform for the generation of B lymphocytes and natural killer cells from human CD34+ cord blood progenitors
Source: Sci Rep. 2025 Dec 18;15:44132. doi: 10.1038/s41598-025-30732-9 (PMC12717158; doi:10.1038/s41598-025-30732-9)
Supplement: Supplementary file 1 — Supplementary Material 1 [file 41598_2025_30732_MOESM1_ESM.docx]

**Supplemental tables**

**Supplemental Table 1. List of materials.**

| REAGENT or RESOURCE | SOURCE | IDENTIFIER |
| --- | --- | --- |
| Antibodies (HSPC Panel) | | |
| 7AAD | Beckman Coulter | A07704 |
| CD10-PC7 | BioLegend | 312214 |
| CD127-R718 | BD Biosciences | 566967 |
| CD135-PE | BioLegend | 313306 |
| CD15-BV510 | BioLegend | 323028 |
| CD34-Pacific Blue | BioLegend | 343512 |
| CD38-BV650 | BD Biosciences | 569966 |
| CD45RA-APCFire750 | BioLegend | 304152 |
| CD7-APC | BioLegend | 982702 |
| CD90-FITC | BioLegend | 328108 |
| Lineage Cocktail-BV510 | BioLegend | 348807 |
| Antibodies (B cell Panel) | | |
| CD10-APCFire750 | BioLegend | 312230 |
| CD127-R718 | BD Biosciences | 566967 |
| CD179α-BV421 | BD Biosciences | 566583 |
| CD19-RB705 | BD Biosciences | 570235 |
| CD34-RB780 | BD Biosciences | 569078 |
| CD38-BV650 | BD Biosciences | 569966 |
| CD45-BV786 | BD Biosciences | 563716 |
| CD79a-PE | BD Biosciences | 555935 |
| FVS660 | BD Biosciences | 564405 |
| IgM-BV510 | BD Biosciences | 563113 |
| TdT-FITC | BD Biosciences | 332789 |
| Antibodies (NK Panel) | | |
| CD10-PC7 | BD Biosciences | 565282 |
| CD122-APC | BioLegend | 339008 |
| CD158a-PE | BD Biosciences | 556063 |
| CD159a-BV786 | BD Biosciences | 747917 |
| CD16-RB705 | BD Biosciences | 570243 |
| CD314 (NKG2D)- BV421 | BioLegend | 320822 |
| CD34-PE/Dazzle594 | BioLegend | 343534 |
| CD38-BV560 | BD Biosciences | 569966 |
| CD3-APCFire750 | BioLegend | 300470 |
| CD45-FITC | Beckman Coulter | 345808 |
| CD56-BV510 | BioLegend | 318340 |
| FVS780 | BD Biosciences | 565388 |
| Antibodies (NK Killing assay) | | |
| PD-L1-PE/Dazzle594 | BioLegend | 329732 |
| B2M-PerCP-Cy5.5 | BD Biosciences | 656645 |
| CD3-PC7 | eBioscience | 25-0038-42 |
| CD56-AF647 | BD Biosciences | 557711 |
| CD107a-APCCy7 | BioLegend | 328630 |
| ULBP2-BV605 | BD Biosciences | 748131 |
| CD314 (NKG2D)-BV786 | BD Biosciences | 743560 |
| Other flow cytometry reagents | | |
| BD Intrasure™ Kit | BD Biosciences | 641778 |
| Brilliant Stain Buffer | BD Biosciences | 563794 |
| Fc Block | BD Biosciences | 564219 |
| VersaLyse lysing solution | Beckman Coulter | A09777 |
| DAPI | Invitrogen | D1306 |
| Critical commercial assays | | |
| CD34 MicroBead Kit UltraPure | Miltenyi Biotec | 130-100-453 |
| CellTrace™ CFSE Cell Proliferation Kit | Invitrogen | C34554 |
| Human Granzyme B DuoSet ELISA | R&D Systems | DY2906 |
| Human IFN-gamma DuoSet ELISA | R&D Systems | DY285B |
| LS Columns | Miltenyi Biotec | 130-042-401 |
| MACS® MultiStand | Miltenyi Biotec | 130-042-303 |
| Pre-Separation Filters (30 µm) | Miltenyi Biotec | 130-041-407 |
| Chemicals and Recombinant Proteins | | |
| Albunorm® 20% | OctaPharma | - |
| Biocoll® (Lymphocytes) | Bio&Sell | BS.L6115 |
| C-Chip (4ch) Hemocytometer | NanoEntek | DHC-NO4 |
| CliniMACS® PBS/EDTA Buffer | Miltenyi Biotec | 700-25 |
| Dimethyl Sulfoxide (DMSO) | Sigma Aldrich | D2650-100ML |
| DMEM | Gibco | 31885-023 |
| Dulbecco’s Phosphate Buffered Saline (PBS), without calcium and magnesium | Sigma-Aldrich | D8537-500ML |
| FCS | Gibco | A5256701 |
| Human Recombinant Flt3/Flk-2 Ligand | STEMCELL Technologies | 78009 |
| Human Recombinant IL-15 | STEMCELL Technologies | 78031 |
| Human Recombinant IL-3 | STEMCELL Technologies | 78040 |
| Human Recombinant IL-7 | STEMCELL Technologies | 78053 |
| Human Recombinant SCF | STEMCELL Technologies | 78062 |
| Pencillin/Streptomycin | Gibco | 15140122 |
| RPMI GlutaMAX | Gibco | 61870036 |
| StemSpan SFEM II | STEMCELL Technologies | 09655 |
| Trypan Blue Stain | Gibco | 15250-061 |
| TrypLE™ Select (1x) | Gibco | 12563-029 |
| Instruments | | |
| Cell-Dyn Ruby | Abbott Diagnostics | 08H67-01 |
| CytoFlex S (V5-B5-R3) | Beckman Coulter | C09734 |
| Software and Algorithms | | |
| CytExpert v2.6 | Beckman Coulter | https://www.beckman.com/flow-cytometry/research-flow-cytometers/cytoflex/software |
| FlowJo v10.8 | FlowJo LLC | http://www.flowjo.com/ |
| GraphPad Prism v8.0.2 | GraphPad Software | https://www.graphpad.com/ |
| Inkscape v1.4.2 | The Inkscape Project | https://inkscape.org/ |
| BioRender Software | BioRender | https://www.biorender.com/ |
| Fiji/Image J | ImageJ | https://imagej.net/ |

**Supplemental Figures**

*
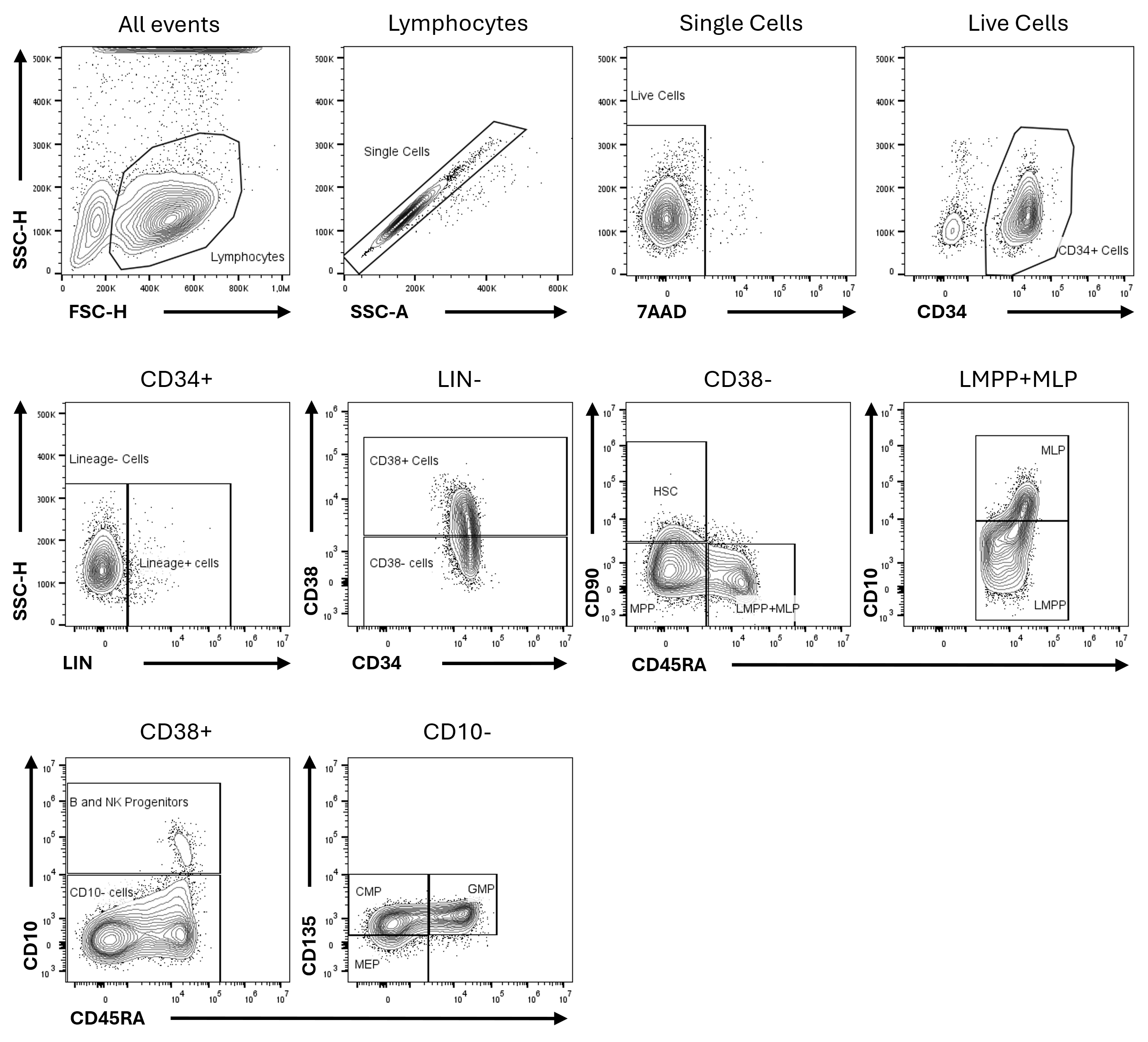
*

**Supplemental Figure 1. Flow cytometric analysis of CD34+ HSPC progenitors during B and NK differentiation.** HSPC Panel successive gating strategy used for flow cytometric identification of HSPC progenitors. Plots are representative of freshly isolated day 0 CB CD34+ cells.

*
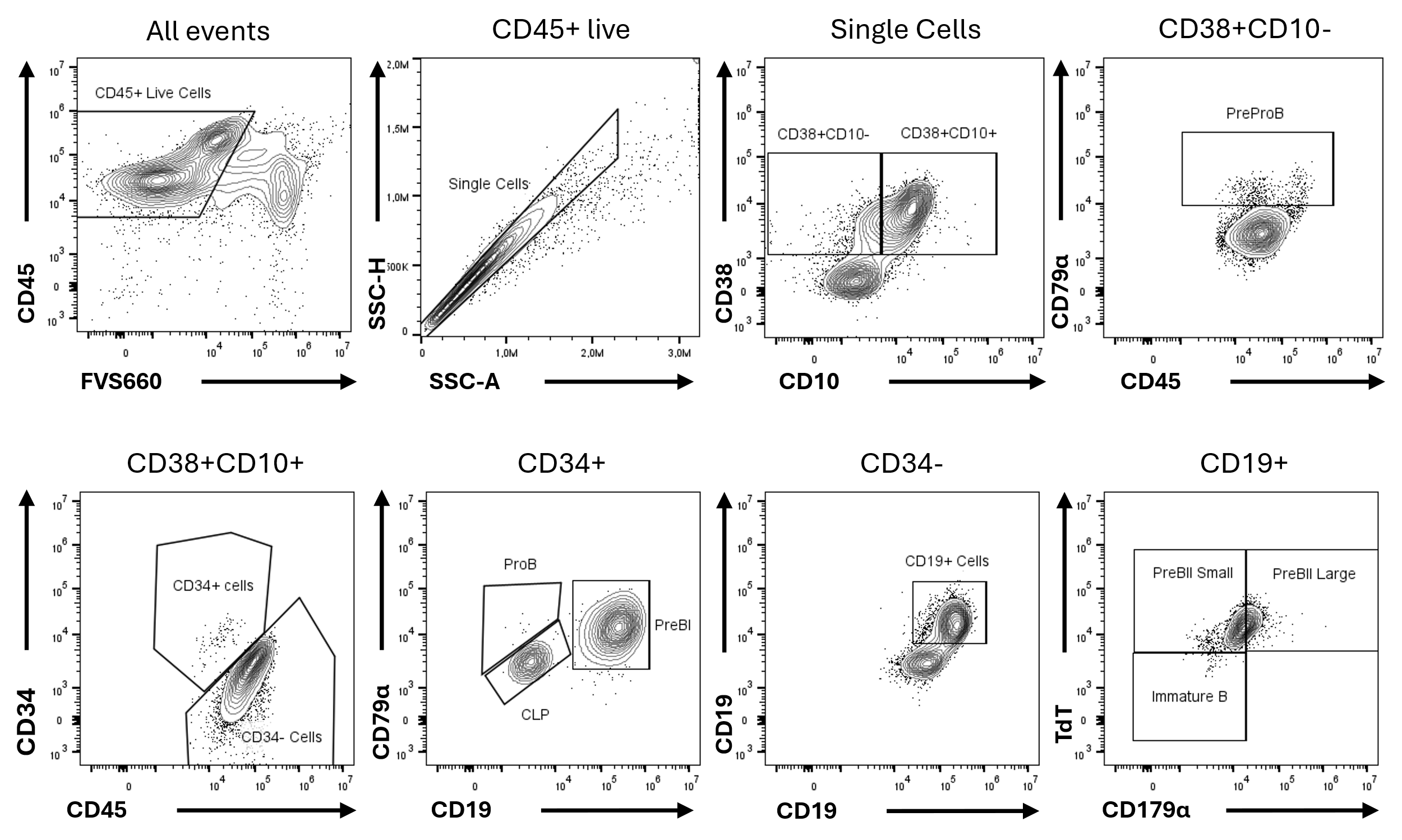
*

**Supplemental Figure 2. Flow cytometric analysis of B lineage progenitors during B cell differentiation.** B cell panel successive gating strategy used for immunophenotypic identification of B lineage progenitors. Plots are representative of cells analyzed on day 35 of B cell differentiation.


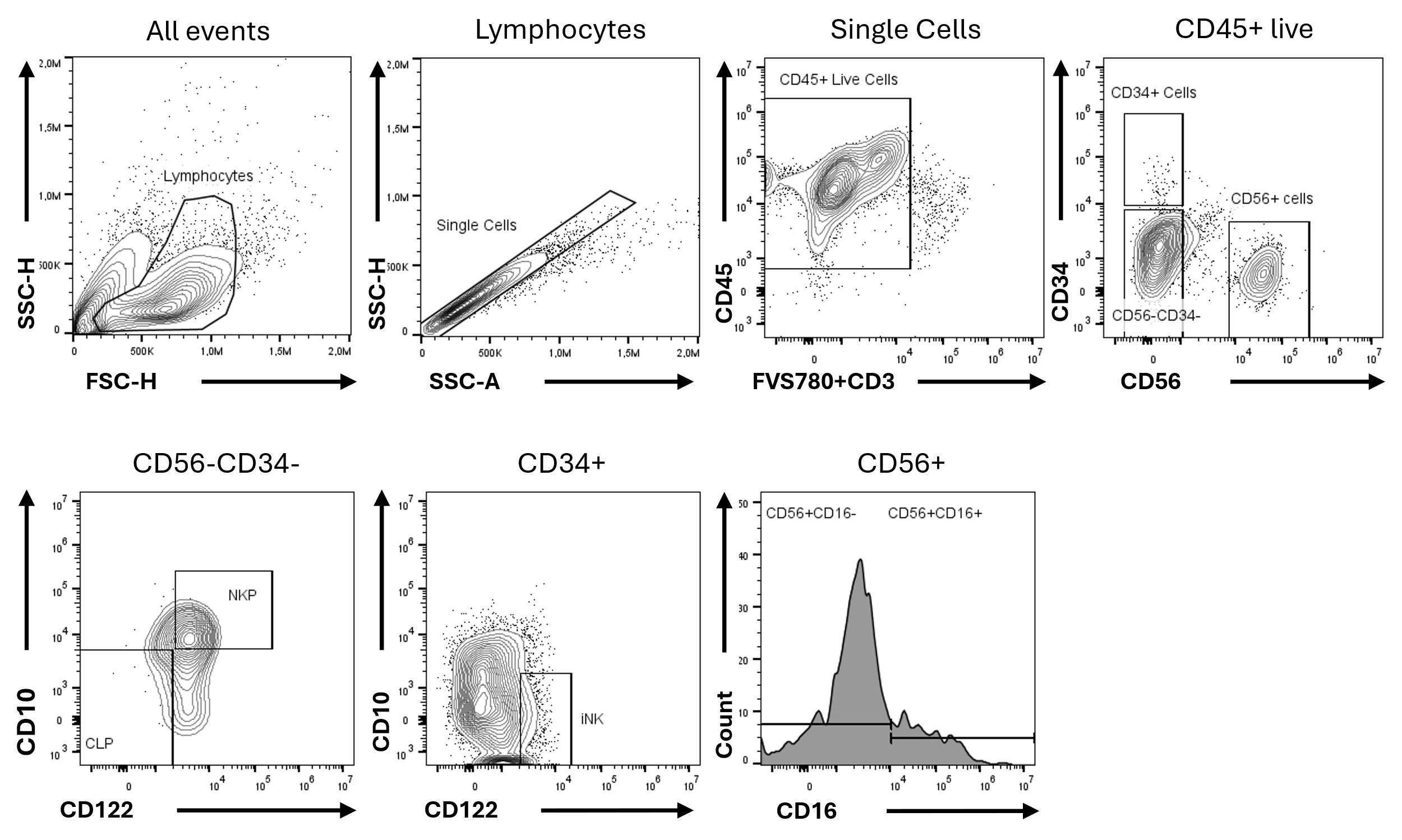


**Supplemental Figure 3. Flow cytometric analysis of progenitor populations during NK cell differentiation.** NK cell panel successive gating strategy used for immunophenotypic identification of NK lineage progenitors. Plots are representative of cells analyzed on day 35 of NK cell differentiation.


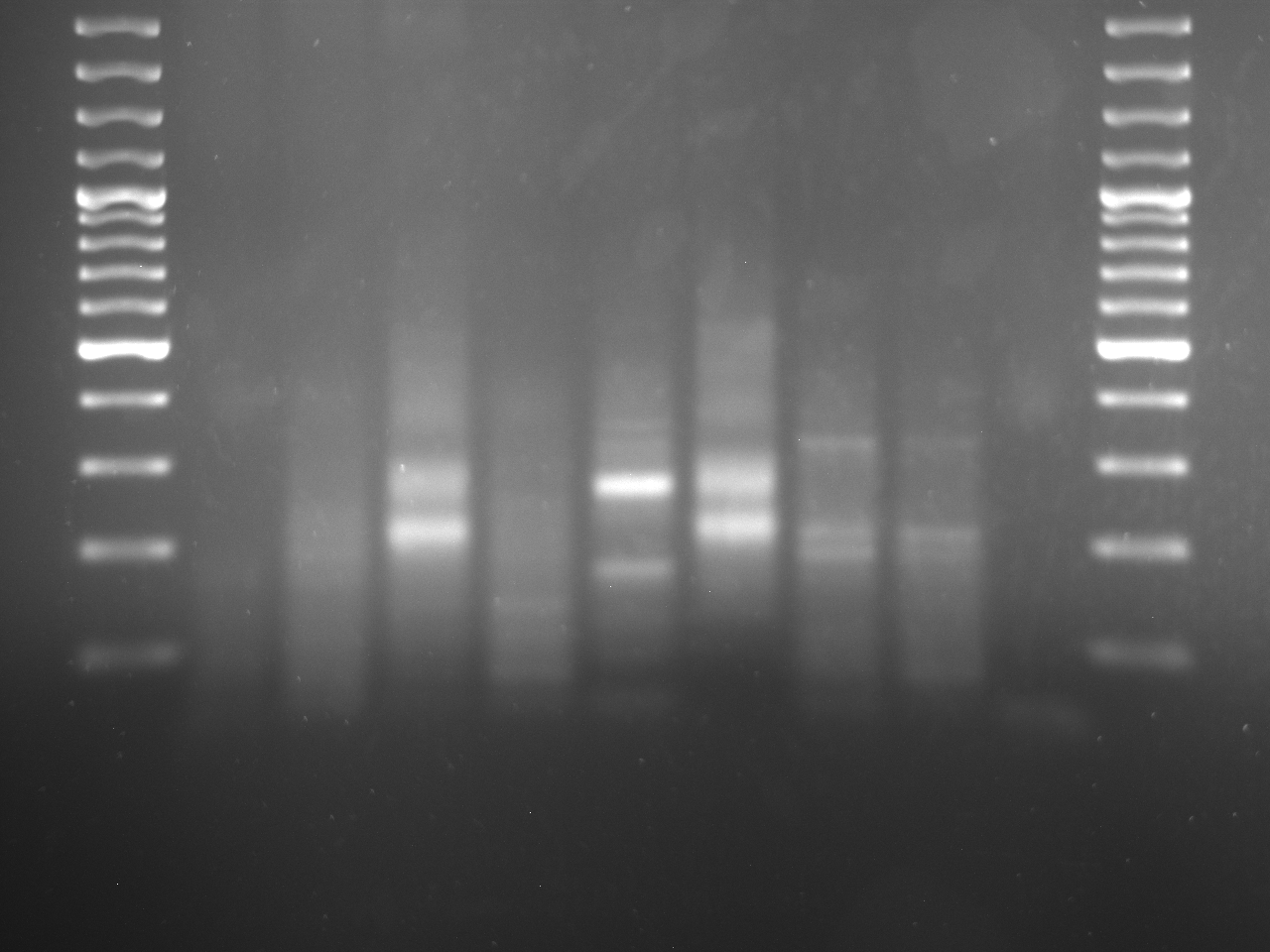


**Supplemental Figure 4. Full, uncropped, and unedited agarose gel corresponding to Figure 3G.** Complete DNA ladders on both sides of the gel are shown (GeneRuler 100 bp Plus DNA Ladder, #SM0321).
